# Next generation agricultural system models and knowledge products: Synthesis and strategy

**DOI:** 10.1016/j.agsy.2017.05.006

**Published:** 2017-07

**Authors:** John M. Antle, James W. Jones, Cynthia Rosenzweig

**Affiliations:** a Oregon State University, United States; b University of Florida, United States; c NASA/Columbia University, United States

## Abstract

The purpose of this Special Issue of *Agricultural Systems* is to lay the foundation for the next generation of agricultural systems data, models and knowledge products. In the Introduction to this Special Issue, we described a vision for accelerating the rate of agricultural innovation and meeting the growing global need for food and fiber. In this concluding article of the NextGen Special Issue we synthesize insights and formulate a strategy to advance data, models, and knowledge products that are consistent with this vision. This strategy is designed to facilitate a transition from the current, primarily supply-driven approach toward a more demand-driven approach that would address key Use Cases where better data, models and knowledge products are seen by end-users as essential to meet their needs.

## Highlights

• High-level insights from the NextGen Special Issue are synthesized.
• A demand-driven strategy to achieve the NextGen vision is formulated.
• The strategy links data, model and knowledge products to Use Cases.

## 1 Introduction

The purpose of this Special Issue of *Agricultural Systems* is to lay the foundation for the next generation of agricultural systems data, models and knowledge products. In the Introduction to this Special Issue, we described a vision for accelerating the rate of agricultural innovation and meeting the growing global need for food and fiber. The use of the new generation of models leads to ‘virtual’ and ‘computational’ agricultural research and development that can complement, and increasingly substitute for, conventional, ‘on-the-ground’ experimental methods. Likewise, significantly improved data and models can contribute to development of advanced farm-management systems, and by making better information available about new systems, could accelerate the adoption and efficient use of more productive and more sustainable technologies. Such data and models are also essential tools for assessing the landscape-scale impacts of technologies, evaluating policies to improve resource management, and projecting the performance of technologies under changing climatic and other environmental conditions.

In this concluding article of the NextGen Special Issue we aim to synthesize insights and formulate a strategy to advance data, models, and knowledge products that are consistent with this vision. As the papers in this Special Issue show, there are many ways that agricultural systems data and models could be improved and linked to new and more-effective knowledge products. Here we focus on the high-level insights that will help inform a strategy for moving the field of agricultural systems science to meet the NextGen goals we have identified – namely, forming the foundation for a computational agricultural science that can move agriculture regionally and globally toward sustainable systems that can meet the food security, nutrition, and health challenges of the 21st Century.

## 2 Closing the gaps between data, models, and knowledge products

### 2.1 The data imperative

Data are the foundation for all science and for agricultural systems analysis. A theme throughout the Special Issue is the need for better data from a wide range of systems and settings. Indeed, better data may be the single greatest need and challenge to achieving the NextGen vision. In the “State of Science” paper, Jones et al. found data limitations to be a common theme among the five Use Cases described in the Introduction to the Special Issue. Jones et al. argue that limitations of current agricultural system models and tools are mainly rooted in inadequate data, and that data limitations are far more important than gaps in basic science understanding and conceptual approaches. Moreover, data limitations restrict predictive capabilities, and this in turn erodes model credibility among users, a key point we return to below. Existing models could address many important Use Cases with better data.

Antle et al. (in this issue) also emphasize the challenge of the need for better data and identify it as a key element of both the ‘pre-competitive space’ of data and model development as well as the ‘competitive space’ of knowledge product development. They describe an approach that would integrate on-farm decision support systems with landscape-scale data and models for policy decision support. Capalbo et al. (in this issue) elaborate an example of this approach using existing models that could be linked with current information technologies. A key issue in implementation of obtaining better data for models is how to create incentives for farmer participation, as well as the participation of the private-sector knowledge product industry that is rapidly advancing.

Data is also a main theme of the paper by Janssen et al. (in this issue). They find a need for both technology and data innovations to meet the needs of the Use Case personas. They describe opportunities to improve data inputs into models through better utilization of existing and new sensor and data collection methods, including remote sensing, crowdsourcing, and mobile technology. They also discuss new tools to generate, archive, access, analyze, visualize, and interpret model inputs and outputs. The paper by Donatelli et al. (in this issue) emphasizes the need for better data to improve the quality and availability of data for pest model inputs and evaluation. They also note that ongoing climate change means that existing data on yield losses from pest damage are becoming obsolete, illustrating the challenges of incorporating non-stationarity into on-going agricultural model development.

The paper by Hwang et al. (in this issue) on incorporating genetic information in crop models highlights the need for a different type of data to improve crop model capabilities. Geneticists and plant breeders typically conduct experiments using a large number of genotypes (e.g., hundreds during their process of germplasm selection for new varieties), while agronomists and crop modelers typically include only a few genotypes combined with different environments and management. Incorporation of genetic information in crop models will enable the models to better simulate interactive genetic, environment, and management effects on production (GxExM), and to do this without having to collect additional data to estimate genotype-specific parameters as is the normal practice now (see White and Hoogenboom, 2010, Messina et al., 2006, Hammer et al., 2006). Characterizing genotype-specific parameters is becoming practical due to the inexpensive and rapid genotyping now widely available to research organizations and the private sector. As pointed out in Hwang et al. (in this issue), data are needed that include a wider range of diverse genotypes combined with a range of environments to develop relationships needed to incorporate gene-based features in crop models. For this reason, extensive collaboration is needed between crop modelers, geneticists, and breeders to design the relevant experiments and obtain appropriate genotype and phenotype response data to use in developing and evaluating the models.

### 2.2 Closing the gap between models and users

The papers in this Special Issue as well as stakeholder consultations by the NextGen author team and by the Agricultural Model Intercomparison and Improvement Project (AgMIP) show that a second key challenge to be addressed is the gap between models and users. This gap manifests itself in several ways.

First, the Use Cases introduced in the Introduction to the Special Issue, as well as many other Use Cases, demonstrate that most users of model outputs do not need or want direct interaction with models; rather, they need to be able to access information in ways and forms that are useful to them. Whether decisions involve farm-level management or high-level policy, it is necessary to take into account the biophysical and economic context in which the relevant systems are operating. Yet, the review of the agricultural systems models in the background papers shows that few, if any, current agricultural systems models are designed to link efficiently to context-specific data and to work with user-friendly applications.

A second gap between models and users identified by stakeholders concerns the credibility of agricultural systems models. Stakeholders emphasize the need for transparent evaluations of agricultural models so that the models will be accepted as part of the evidence base for making farm management decisions and for policy formation. To achieve this goal, several papers in this Special Issue emphasized the need for context-specific analysis to provide evidence of model capabilities and evaluation of model uncertainty. Quantification and communication of uncertainties related to data, parameterizations, and scenarios can contribute to building confidence in agricultural models.

Third, many users now need to know more outcomes than crop yields alone. The goal of assessing system sustainability has extended the scope to include economic and environmental outcomes, as well as other consequences for human well-being such as nutrition and human health. These dimensions add even greater data demands, and are hampered by the less-developed state of quantitative models for some outcomes such as nutrition and health. As a result, current modeling studies often resort to ad hoc procedures, such as using simple empirical models when process-based models are not available.

Spatial and temporal scales are also related to relevance. Detailed studies of “representative farms” or “case studies” cannot be credibly used as the basis for policy decision-making that necessarily must consider larger geographic regions and significant proportions of human populations. Designing comprehensive ‘systems’ models remains a major challenge to be addressed by ‘out-scaling’ of better inter-operable model components.

## 3 NextGen strategy

Our strategy for the next generation of data, models, and knowledge products is based on the insight that to achieve the NextGen vision, agricultural system research must transition from a primarily supply-driven (i.e., science-driven) approach, toward a more demand-driven (i.e., end-user or stakeholder-driven) approach. This re-orientation is necessary to guarantee that the key gaps identified above will be addressed, and it is needed to garner financial support for investments in NextGen data, models and knowledge products. In our estimation, NextGen developments for agricultural systems will not be supported primarily through conventional funding for basic or applied science. Rather, the needed investments increasingly will be made to address key Use Cases where better data, models and knowledge products are seen by the ultimate users as essential to meet their needs. We expect these investments to be made by both public and private-sector organizations willing to support important Use Case applications.

**Fig. 1:**
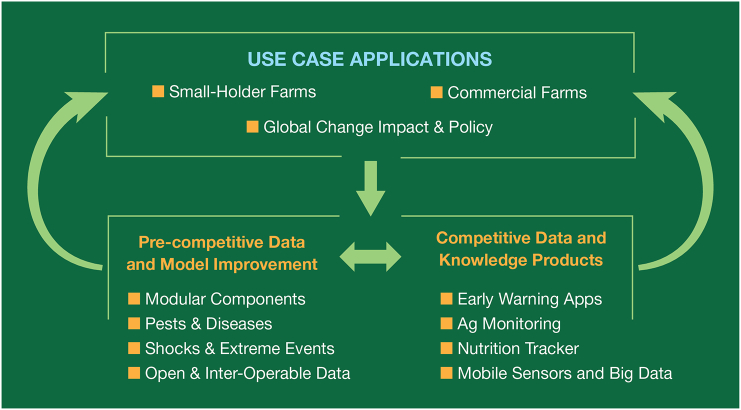
Components of a strategy for NextGen data, model and knowledge product developments to support key use case applications.

These investments in NextGen should be implemented through an elaboration and extension of AgMIP's two-track science approach (Rosenzweig et al. 2013) that incorporates the key insights outlined above. The two-track science concept is based on parallel and coordinated activities that involve: (1) improvement of data and models through systematic inter-comparison, improvement and integration; and (2) application of models to important Use Cases. NextGen adds to this two-track science concept the recognition that there are two ways that these developments can be made, i.e., in the “pre-competitive” space of data and science developments that are public goods, as well as in the “competitive space” of knowledge product developments that are largely private goods, but that can also provide important data as well as insights into what elements of model development are most useful to decision-makers. Thus, by combining AgMIP's emphasis on both science and application of data and models with NextGen insights, we arrive at a new conceptual model for data and model improvement and application that involves both pre-competitive elements and competitive elements, with all of these interacting and overlapping to some degree (Fig. 1).

We see three types of Use Cases that will provide the basis for NextGen advances. The first corresponds to the Use Cases 1–3 described in the Special Issue introduction, and addresses investments designed to improve smallholder farming systems in the developing world. Some of the demand for knowledge products to support these kinds of Use Cases will come from traditional governmental development agencies, but increasingly we expect the demand will come from private entities, both charitable organizations and agribusiness. The second type of Use Cases involves management of commercial farming systems. We expect these developments will be funded largely through private-sector organizations that provide management services and inputs to farmers. A third type of Use Case is for global change research. These Use Cases are largely funded by public sector institutions but also increasingly by private donors. One important Use Case in this domain is the Coordinated Global and Regional Assessment program being developed by AgMIP (Rosenzweig et al., 2016). The data, model and knowledge product developments for these three types of Use Cases will have substantial overlaps and complementarities, and so it will be important for all of them to interact with both the pre-competitive and competitive spaces as they are implemented.

### 3.1 NextGen for smallholder farms in the developing world

Innovations in models and knowledge products are happening across the spectrum of agricultural systems, but many of the recent innovations in ICT have been focused on commercial-scale systems in the United States and other high-income countries. There is a clear need for pre-competitive public good investments to develop data, models, and knowledge products suitable for smallholder agricultural systems in the developing world. Corresponding to the design of Fig. 1, the strategy should facilitate the growth of communities of science and practice in both the pre-competitive and competitive dimensions.

AgMIP and allied organizations should continue to play a lead role as conveners for the science community and to advance the agricultural systems science and data integration capabilities, with close collaboration with the knowledge product community. AgMIP would work with donors to develop funding mechanisms to support this component, including support from the foundations and other donors, public institutions, and public-private partnerships. Private-public partnerships among research, donor, and private organizations should collaborate to lead the development of the community of knowledge product developers and users, along with development of funding mechanisms that involve public institutions, private donors, and the private-sector technology and agri-business communities.

These two communities need to interact closely to ensure that the design of the NextGen data, models and knowledge products for smallholder farmers are closely connected to and driven by the key Use Cases identified by the user community. To move forward, NextGen pilot projects could focus on major smallholder farming systems in various regions at both farm and landscape scales. The goal of the pilot projects would be to develop knowledge products, and the improved model components and ICT platforms needed to support them. To ensure their relevance and to help obtain financial support, the NextGen activities should be linked to major donor projects, thus embedding the development of new technologies into the ongoing work. Work in both pre-competitive and competitive spaces should be coordinated, and should involve teams of scientists, ICT specialists, and application developers.

### 3.2 NextGen for commercial agriculture

A long-term strategy for next generation models and knowledge products used in commercial agriculture and agribusiness could be to encourage developments on both the demand side and the supply side of the ‘market’. To date, most model development has come from the supply side (i.e., basic and applied, publicly-funded research), and there is a particular need to develop the demand side with involvement by private and public institutions, the science community, and end-users. Knowledge product developments should be linked with improved engagement of traditional end-users, including both small-holders in the developing world as noted above, and larger-scale commercial agriculture and farm management advisors, as well as policy decision-makers across the spectrum from local to national and global (as discussed further below). There is also a need to engage new potential end-users, including private-sector firms engaged in providing management advisory services, as well as the crop insurance and reinsurance industries.

On the supply side, we see a role for private-public partnerships to facilitate data collection and sharing, as well as collaborative model development and testing, combined with better communication with the demand side to help guide the researchers in the pre-competitive space toward the model developments that could be useful in the competitive space of knowledge products. Capalbo et al. (in this issue) provide an example of how this could be implemented.

The concept of competitive space is typically conceived as the development of knowledge products that are provided through commercial markets – i.e., as “private goods.” A key challenge for our vision is how to incentivize and stimulate this demand side activity and facilitate its linkages to the more traditional research-based supply side. We think that a key element that ties the demand-side and supply-side together is the common need for data, as illustrated in Jones et al. (in this issue), Antle et al. (in this issue), Janssen et al. (in this issue) and in Fig. 1. The development of new data infrastructure will play a key role in how agricultural systems science evolves, and will be closely linked to the development of the pre-competitive and competitive spaces.

It is clear that a new data infrastructure is emerging in the United States and other countries where commercial agriculture is rapidly increasing its use of information technology to generate and use “big data.” A key feature of this emerging system is that it must involve both private and public data (Antle et al., in this issue). The acquisition, management, and use of each type of data bring critical challenges that must be addressed if data are to be open and usable for both research and decision support.

Provision of data by private entities for public uses can be based on voluntary participation by private entities, a mandatory system, or a combination of the two. A voluntary system is likely to be more politically and socially acceptable, and can generate quality data if participants are motivated to provide accurate information. Creating a system with clear benefits to all parties would facilitate voluntary participation. For example, value could be in the form of management tools and data that improve a farm's economic and environmental performance, and also provide data for product quality certification or regulatory compliance, as in Capalbo et al. (in this issue). Another approach could be to provide financial compensation for the participants' time spent in creating the new datasets. The costs of a voluntary system could be covered, at least in part, from savings realized by reducing the use of paper-based survey instruments and enumerator interviews.

There are various challenges to the implementation of a voluntary approach to data sharing. First, it may not be possible to achieve the needed statistical representation of all regions and farm types needed for comprehensive research and policy analysis. One way to ensure adequate representation would be to combine a voluntary system with compensation or other incentives for participation in regions or systems with sparse data. Another strategy would be to require farmers in voluntary government subsidy, conservation, or environmental payment programs to participate in the data system, as is currently done in many countries, to varying degrees.

Privacy concerns are another consideration in developing data infrastructure, and have been the subject of recent discussions among farmers and commodity organizations in the United States and other countries as they explore the use of new technologies and big-data analytics. Technological solutions to these concerns exist – for example, the online financial transaction systems now in widespread use show that data can be securely transmitted and stored electronically. But other challenges, such as addressing confidentiality requirements for public data, will require legal and institutional innovations.

We envisage private-public partnerships to advance the development of a massive new data infrastructure that can advance both private and public interests. Much of the data needed for a new system is already being collected by individuals, governments, and private companies, and innovative initiatives are demonstrating the feasibility of acquiring, storing, and using data securely and efficiently (Antle et al., in this issue). Currently, both private and public entities are engaged in development of technology and software for collecting and storing data and for developing analytical tools.

The large number and rapid evolution of these advances makes coordination a major challenge. One solution appears to be a partnership among the organizations that have a mutual interest in assuring that the data are obtained efficiently and used appropriately for both private and public purposes. A private-public partnership for a new data infrastructure could be supported by various stakeholder organizations, including producer and industry organizations, agricultural commodity organizations, and non-governmental organizations. An example in the UK is a joint industry and academic initiative, the Centre for Agri-Informatics and Sustainability Metrics (AIMS).

Collaboration with research organizations promoting better public data such as AgMIP and scientific societies will also be needed to provide leadership and coordination. A critical issue is how long-term funding for the creation and maintenance of the data and knowledge infrastructure will be achieved. While short-term research funding can make an important contribution, on-going support will be needed to create and maintain the data system. For example, the Global Open Data for Agriculture and Nutrition initiative (GODAN, www.godan.info) is a collaboration of 120 partners from national governments, non-governmental, international and private sector organizations that have committed to harness the growing volume of data for users working in agriculture and nutrition. This type of ‘Bretton Woods’ organization is essential for ongoing coordination.

A coordinated pilot program funded through a private-public partnership could develop and test innovative approaches to incentivize data sharing and facilitate data acquisition, management, storage and utilization. Modular, inter-operable public-domain software models and knowledge products linked to them could be made available to users who agree to share data. These models and knowledge products could be linked to a cloud-based data retrieval and storage system, such as the one being developed by private data programs like On-Farm Network and public ones like AgMIP's activities coordinated with the U.S. Department of Agriculture and other organizations.

### 3.3 NextGen for global change research

**Fig. 2:**
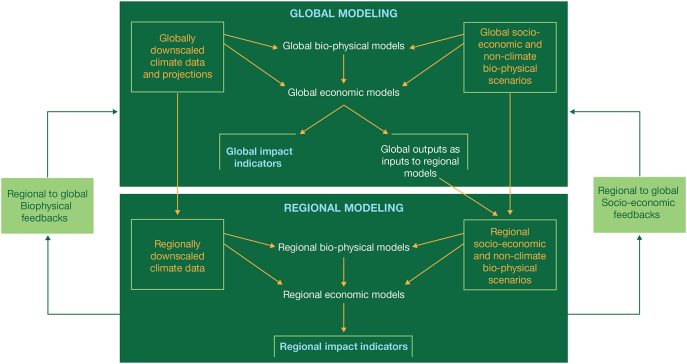
Global and regional model components of the AgMIP Coordinated Global and Regional Integrated Assessment (CGRA).

Global change research on the potential agricultural impacts of global climate change, as well as research on possible mitigation and adaptation strategies at global and regional scales, have relied heavily on the use of agricultural system models. AgMIP has developed a plan to substantially improve the science basis and relevance of such assessments through a new Coordinated Global and Regional Assessment program (Rosenzweig et al. 2016). This plan links global integrated assessment data, scenarios, and models with regional assessments, following a set of protocols similar to the ones developed recently by AgMIP (Rosenzweig et al., 2015). Fig. 2 shows the structure of the global and regional assessments and how they can be linked. As Fig. 2 shows, agricultural system models play a key element in both global and regional assessments. At the global scale, globally gridded crop and livestock models simulate the impacts of climate change, mitigation and adaptation on crop and livestock productivity, and these data then become inputs into global economic models that simulate key economic and food security indicators such as per capita food consumption, food prices, and food trade. At the regional scale (meaning national or within-country levels), global model projections of prices are combined with the outputs of agricultural system models and used as inputs into more detailed economic impact assessment models. The agricultural system models are simulated at field or farm scales with more detailed soils, climate and management data than is possible at the global scale, and at multiple locations to represent the large degree of heterogeneity in agricultural systems that is observed across agricultural landscapes. These models simulate climate, mitigation and adaptation impacts on crop and livestock productivity that are used in more detailed economic impact assessment models.

It is now widely recognized that food production is a necessary but not sufficient condition for food security at global and regional scales, and that there is a substantial potential for global climate change to disrupt food systems around the world (Brown et al. 2015). Attempts to assess these potential disruptions, such as the Intergovernmental Panel on Climate Change (IPCC) Assessment Reports, have been hampered due to inconsistencies in methodologies, assumptions, models, scales, regional coverage, and transparency in agricultural impact studies.

To respond to these challenges, the Aspen Global Change Institute convened the AgMIP Workshop on Coordinated Global and Regional Integrated Assessments (CGRA) of Climate Change and Food Security September 13–18, 2015. The workshop brought together 45 disciplinary and international agricultural experts to draft protocols and implementation plans for the CGRA, which will contribute to the IPCC Sixth Assessment Report. The assessments will cover global-scale modeling of crops, livestock, and economics, linked to major agricultural regions in North America, South America, Europe, Africa, East Asia, and Australia, enabling characterization of climate impacts on both large-scale and smallholder farming systems.

**Fig. 3:**
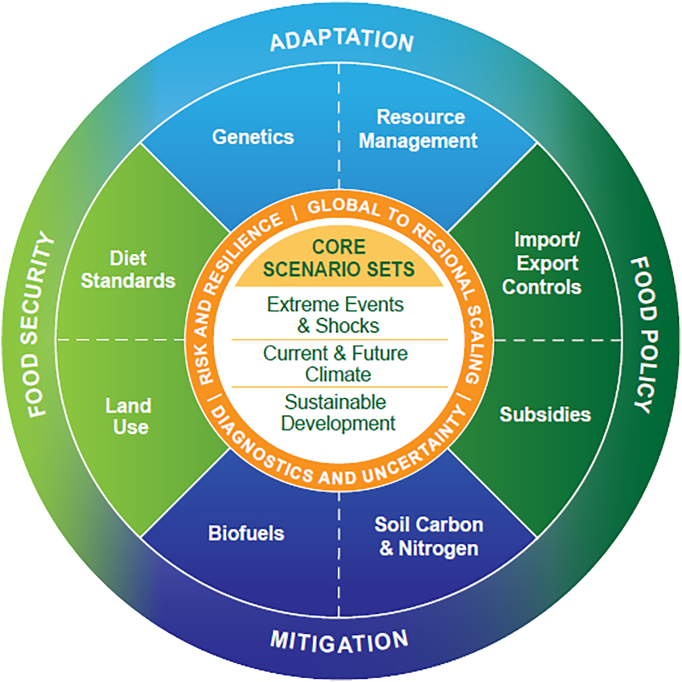
Key elements of the AgMIP Coordinated Global and Regional Assessment framework.

Based on discussion with the stakeholders in attendance at the workshop, three advances in defining the foci of the CGRA occurred at the Aspen Workshop (Fig. 3). These advances are a focus on *risk management*, on *current as well as future* time periods, and on *nutrition* outcomes. Improved agricultural modeling can enable the development of risk management strategies that are effective even under changing climate. A focus on both current and future time periods links the assessment to real-time decision-making as well as planning for future food challenges. Finally, the focus on nutrition outcomes bridges the gap between agricultural outputs and human well-being. Adaptation, mitigation, food security, and food policy are the main focal areas for CGRA analysis (Fig. 3).

(Source: Rosenzweig, Antle, and Elliot, 2016.)

The protocol-based CGRA promises to substantially improve the analytical consistency of global and regional assessments, and improve the characterization of data, model and scenario uncertainties associated with projections of agricultural and food security impacts for IPCC AR6 and other stakeholders. CGRA protocols will feature consistent multi-model, multi-discipline, and multi-scale assessments of climate impacts on agriculture and food security. Simulations will be rooted in historical observations and climate projections of both means and extreme events from the most recent global climate model simulations, and will be driven by scenarios linking global emissions pathways with global and regional socio-economic scenarios. A key coordination mechanism will be Representative Agricultural Pathways (RAPs) developed at both global and regional scales (Valdivia et al. 2015). RAPs are designed to be part of a logically consistent set of drivers and outcomes from global to regional and local.

CGRA results will have direct implications for international climate policy, regional adaptation and mitigation planning, and development aid. CGRA outputs will be available to inform and improve integrated assessment modeling, nitrogen and carbon cycle simulations, and impact projections of land use, water resources, ecosystems, and urban food supply.

## 4 Concluding observations

The Introduction to this Special Issue began with our observation that there is a growing demand for agricultural systems data, models, and knowledge products to be used to support decision-making from farm to regional to global scales. One example of this need is illustrated by the agreement reached at the United Nations 21st Conference of the Parties held in Paris in December 2015. Countries agreed to make commitments to hold the increase in the global average temperature to well below 2 °C above pre-industrial levels and to pursue efforts to limit temperature increase to 1.5 °C. As part of the agreement, the Conference of the Parties requested the Intergovernmental Panel on Climate Change to prepare a Special Report on the mitigation efforts that would be needed to achieve these goals, to be published in 2018.

Providing such a report on this timeline is a major scientific challenge, and highlights the need for the scientific community to be able to respond in a timely manner to the public's need for information convened in ways that can be readily accessed and used. Assessments of agriculture's potential to mitigate greenhouse gas emissions, and the evaluation of impacts on food security at global and regional scales, are just two ways that agricultural systems models will need to be used in addressing this challenge. We conclude by observing that NextGen data, models and knowledge products envisioned in this Special Issue would provide the capability to provide better answers to these questions, and to other important questions that depend on a significantly improved understanding of agricultural systems from farm to regional and global scales.
